# Draft Genome Sequence of *Pseudomonas* sp. Strain BMS12, a Plant Growth-Promoting and Protease-Producing Bacterium, Isolated from the Rhizosphere Sediment of *Phragmites karka* of Chilika Lake, India

**DOI:** 10.1128/genomeA.00342-16

**Published:** 2016-06-30

**Authors:** Samir R. Mishra, Ananta Narayan Panda, Lopamudra Ray, Neha Sahu, Gayatri Mishra, Sudhir Jadhao, Mrutyunjay Suar, Tapan Kumar Adhya, Gurdeep Rastogi, Ajit Kumar Pattnaik, Vishakha Raina

**Affiliations:** a School of Biotechnology, KIIT University, Bhubaneswar, Odisha, India; b Wetland Research and Training Center, Chilika Development Authority, Department of Forest and Environment, Bhubaneswar, Odisha, India; c Bionivid Technology Private Limited, Kasturi Nagar, Bangalore, India

## Abstract

We report the 4.51 Mb draft genome of *Pseudomonas* sp. strain BMS12, a Gram-negative bacterium in the class of Gammaproteobacteria, isolated from the rhizospheric sediment of *Phragmites karka*, an invasive weed in Chilika Lake, Odisha, India. The *Pseudomonas* sp. strain BMS12 is capable of producing proteases and is also an efficient plant growth promoter that can be useful for various phytoremedial and industrial applications.

## GENOME ANNOUNCEMENT

*Pseudomonas* species are Gram-negative, rod-shaped, aerobic, nonfermenting, nonspore forming, motile, catalase positive, oxidase positive bacteria that belong to the class of Gammaproteobacteria and are common inhabitants of soil and water. Recent studies have shown that *Pseudomonas* sp. is useful as a microbial inoculant for the degradation of a diverse range of compounds including polycyclic aromatic hydrocarbons (1). It has also been known to provide antibiotic resistance, implying a clinical importance (2). On the basis of 16S rDNA gene sequence analysis, *Pseudomonas* sp. strain BMS12 was classified in the genus of *Pseudomonas* (3). However, little is known about the plant growth-promoting factors associated with *Pseudomonas* spp. (4). Chilika is the biggest lagoon along the east coast of India, situated between latitudes 19°28′ and 19°54′ N and longitude 85°05′ and 85°38′ E (5). *Phragmites karka* (a common reed) forms monoculture dense patches in Chilika Lake and is considered a highly invasive weed (6).

The microbial communities associated with the rhizospheric region of these macrophytes contributes to overall biogeochemical cycling, and thus play an important role in nutrient cycling in the lagoon ecosystem. The bacterial community associated with the weeds produce many plant growth-promoting compounds, which facilitates the growth of macrophytes in estuarine environments (6, 7). In this study, we report the draft genome sequence of the plant growth-promoting and protease-producing *Pseudomonas* sp. strain BMS12, isolated from the rhizospheric sediment of *Phragmites karka* located at the Bhaseramundia sampling station of Chilika Lake (19°78224′ N and 85°30041′ E), using the dilution plating technique at 30°C in Zobell’s marine broth (ZMB) (HiMedia, India). The strain BMS12^T^ is a Gram-negative, protease-producing bacterium with optimal growth condition at temperature 30°C and pH 7.2

Genomic DNA was extracted using the GNOME kit (MP Biomedicals, Santa Ana, CA, USA). The genome sequence of strain BMS12 was sequenced using an Illumina MiSeq sequencing platform. The data generated was assembled using Velvet (v1.2.10.) (8), resulting in 81 contigs, a total of 4,513,937 bp, and a *N*_50_ contig size of 233,632 bp. The estimated complete genome size was 4.51 Mb, with a G+C content of 63.94%. Genome annotation was performed using the Rapid Annotation using Subsystems Technology (RAST) (3, 9), which predicted a total of 4,128 protein coding sequences, 56 pseudogenes, 53 tRNAs, and 3 rRNA clusters. The taxonomy identification was performed using EzTaxon and MEGA6, which identified *Pseudomonas* sp. strain BMS12 as the putative species per sequence homology of the assembled genome.

Genes for plant growth-promoting characteristics and protease degradation are present, corroborating results from RAST annotations that identified various gene clusters for 1-aminocyclopropane1-carboxylate deaminase activity, auxin biosynthesis, nitrogen, metabolism, siderophore production, phosphorous solubilization, various antibiotic resistance gene clusters, protein degradation, degradation of aromatic compounds, stress regulation genes, poly-β-hydroxybutyrate (PHB) metabolism, lipid metabolism, and sulfur metabolism. In conclusion, *Pseudomonas* sp. strain BMS12 is a promising candidate as an inoculant to stimulate phytoremediation and also for protease enzyme production.

### Nucleotide sequence accession numbers.

This whole-genome shotgun project has been deposited in DDBJ/EMBL/GenBank under the accession no. LSOF00000000. The version described in this paper is the first version, LSOF01000000.
